# Chimeric Fimbrial Multiepitope Antigen Fused to Double-Mutant LT (dmLT) Induces Antibodies That Inhibit Enterotoxigenic *E. coli* Adhesion in Porcine IPEC-J2 Cells

**DOI:** 10.3390/ani15192858

**Published:** 2025-09-30

**Authors:** Jinxin He, Hongrui Liu, Yuexin Li, Jiashu Chang, Yayun Yang, Shaopeng Gu

**Affiliations:** College of Veterinary Medicine, Shanxi Agricultural University, Jinzhong 030801, China

**Keywords:** enterotoxigenic *Escherichia coli*, fimbriae, double-mutant heat-labile toxin, multiepitope fusion antigen, vaccine

## Abstract

This study focused on utilizing the double-mutant heat-labile toxin (R192G/L211A) (dmLT) as a backbone protein, into which neutralizing epitopes of ETEC (FaeG, FedF, FanC, FasA, and Fim41a) were embedded. A combination of computational modeling and immunogenicity analysis was conducted to evaluate the dmLT_(R192G/L211A)_ multiepitope fusion antigen (MEFA). Both the computational modeling and experimental results confirmed that all relevant epitopes were clearly exposed on the surface of the MEFA. Subcutaneous immunizations of rabbits with the MEFA protein yielded the development of IgG antibodies that targeted all five fimbriae. Furthermore, these antibodies demonstrated significant inhibition of adhesion for K88+, K99+, 987P+, F18+, and F41+ ETEC strains to porcine small intestinal epithelial cell line IPEC-J2 cells. These results indicated that the dmLT toxoid-based MEFA protein effectively elicits high-titer, functional antibodies capable of neutralizing the attachment of multiple prevalent ETEC fimbrial types, highlighting its potential as a broad-spectrum vaccine candidate. Consequently, it shows promising potential as a broad and effective vaccine against ETEC.

## Simple Summary

Enterotoxigenic *E. coli* (ETEC) causes severe diarrhea in piglets, leading to major economic losses. The currently available vaccines offer limited protection. This study developed a broad-spectrum vaccine candidate using a multiepitope fusion antigen (MEFA) based on the dmLT toxoid backbone, displaying key epitopes from five ETEC fimbriae (FaeG, FedF, FanC, FasA, Fim41a). Computational modeling confirmed proper epitope exposure. In rabbits, the MEFA induced strong IgG responses against all five fimbriae. These antibodies significantly reduced ETEC adhesion to piglet intestinal cells. The results showed the dmLT-MEFA effectively generates neutralizing antibodies against multiple ETEC types, demonstrating its potential as a cross-protective vaccine. This approach could improve piglet health, reduce antibiotic reliance, and benefit the swine industry by preventing widespread ETEC infections through a single, effective immunization strategy.

## 1. Introduction

Enterotoxigenic *Escherichia coli* (ETEC) strains are a leading cause of diarrhea in neonatal and post-weaning piglets, contributing to substantial economic losses in the global swine industry due to increased morbidity, mortality, reduced growth rates, and treatment costs [1]. These strains produce various types of fimbrial or non-fimbrial adhesins, which enable the bacteria to attach host receptors in small intestines for colonization. Additionally, they secrete heat-labile toxin (LT) and heat-stable toxin (ST), which are delivered to small intestinal epithelial cells, disrupting ion and fluid homeostasis and triggering excessive fluid secretion, ultimately leading to watery diarrhea [2]. The effectiveness of antibiotics for treating ETEC-associated diarrhea is diminishing due to the alarming increase in antimicrobial resistance among ETEC strains [3,4]. Consequently, vaccination is widely regarded as the most practical, sustainable, and effective strategy for preventing ETEC-induced diarrhea, particularly in high-risk populations such as neonatal and weaned piglets [5]. However, a major challenge in developing effective ETEC vaccines is the immunological heterogeneity of these strains [6,7]. A basic requirement of ETEC vaccine should induce host immunity to prevent colonization by the most common strains.

ETEC strains have the ability to produce multiple adhesins that facilitate their attachment to the small intestines of their hosts. As a result, these adhesins have traditionally been the main focus in the development of ETEC vaccines [8,9]. While incorporating antigens for all ETEC adhesins may be impractical, a more feasible strategy is to develop a vaccine that provides broad cross-protection against the predominant ETEC strains responsible for the majority of clinical cases [10,11]. This strategy has led to the development of whole-cell oral vaccines, in which several live attenuated or killed ETEC strains are combined in the forms of cocktail vaccines, with the aim of achieving broad protection against the various adhesins [12,13,14]. However, the process has been hindered by its complicated formulation and high cost.

To address the challenge of heterogeneity in ETEC strains and facilitate the development of a safe and cross-protective vaccine, a broad-spectrum approach is needed. The innovative multiepitope fusion antigen (MEFA) vaccinology platform, which is based on epitopes and protein structures, offers a solution by allowing the creation of safe and effective multivalent vaccines [15,16]. The MEFA platform leverages computational biology and protein modeling techniques to rationally design a single immunogen that displays multiple protective epitopes derived from diverse pathotypes and virulence factors, enabling a targeted and broad immune response. This approach preserves the native antigenicity of the epitopes, enabling the design of a safe chimeric protein that effectively elicits broadly protective antibodies against multiple ETEC strains. Thus, the MEFA platform facilitates the development of safe and cross-protective multivalent vaccines [17]. Several backbone proteins, such as FaeG, FedF, and IpaD, have been explored in embedding virulence neutralizing epitopes to create MEFAs capable of inducing antibodies against multiple pathogens [18,19,20]. However, it is important to note that recombinant protein-based vaccines often have limited antigenicity compared to live attenuated and inactivated vaccines, as they lack immune danger signals. Therefore, measures should be taken to necessitate the use of an adjuvant to promote an adequate immune response.

One protein adjuvant that has been studied for antigen delivery is the double-mutant LT (R192G/L211A), also known as dmLT. It has been shown to enhance both serum and mucosal antibody responses [21,22]. Additionally, in a pig model, 3×STaN12S-dmLT has been found to induce protective antibodies against STa+ ETEC diarrhea after intramuscular injection [23]. Furthermore, the MEFA vaccine CFA/I/II/IV-3×STa_N12S_-dmLT has been shown to elicit broad-spectrum immune responses in both mice and pigs, inducing significant anti-adhesion and anti-toxin antibodies following intraperitoneal immunization in mice and intramuscular vaccination in pigs. The resulting serum antibodies effectively inhibited the adhesion of seven major colonization factor antigens (CFA/I and CS1–CS6) and demonstrated potent neutralization of both heat-labile and heat-stable enterotoxins, highlighting the vaccine’s ability to confer comprehensive protection against diverse ETEC virulence factors [24]. In another study, dmLT was fused with PcrV and PopB, proteins from Pseudomonas aeruginosa. The resulting dmLT-PcrV-PopB fusion protein was able to activate dendritic cells in vitro and elicited robust IgG and IgA antibody titers in mice following intranasal administration, demonstrating its immunogenicity and potential as a mucosal vaccine candidate [25]. However, there is limited knowledge on using dmLT as a backbone protein to incorporate virulence neutralizing epitopes. In this research, dmLT was used as a backbone to integrate neutralizing epitopes from adhesive subunits of FaeG, FanC, FasA, FedF, and Fim41a. By integrating structural vaccinology, epitope-focused design, computational biology, and protein modeling, the dmLT-based fimbrial MEFA platform enables the precise display of multiple protective epitopes from diverse ETEC virulence factors on a single immunogenic backbone, while preserving their native antigenic conformation to elicit targeted and broad immune responses. The MEFA protein has proven effective in eliciting neutralizing antibodies that significantly block the adhesion of K88+, K99+, 987P+, F41+, and F18+ ETEC strains to porcine intestinal epithelial cells, demonstrating its potential to confer broad protection against major ETEC fimbrial types. Overall, these results suggest that dmLT fimbriae MEFA could serve as an effective and broad-spectrum vaccine candidate for K88+, K99+, 987P+, F41+, and F18+ ETEC.

## 2. Materials and Methods

### 2.1. Bacterial Strains and Cell Line Culture Conditions

The porcine ETEC strains expressing K88, K99, 987P, F18, or F41 fimbriae were obtained from the China Institute of Veterinary Drug Control (CIVDC) and used in in vitro adhesion inhibition assays to evaluate the functional activity of porcine ETEC antibodies. The expression vector pET22b (Novagen, Madison, Wisconsin, USA) and *E. coli* strains DH5α and BL21 (DE3) were used for gene cloning and recombinant protein expression. The porcine intestinal epithelial cell line IPEC-J2 was cultured in Dulbecco’s modified Eagle’s medium (DMEM) supplemented with 10% heat-inactivated fetal bovine serum (FBS) (Gibco) and maintained at 37 °C in a humidified atmosphere containing 5% CO_2_. Chicken IgY antibodies against recombinant FaeG, FanC, FasA, FedF, and Fim41a were previously made in our lab. The cell line of T-84 and IPEC-J2 were kindly provided by Dr. Zhenyu Wang at Anhui Agricultural University. The 3D structural modeling was performed using I-TASSER, a widely used platform for protein structure prediction and conformational sampling.

### 2.2. Construction of dmLT MEFA

The neutralizing epitopes of K88+, K99+, 987P+, F41+, and F18+ETEC fimbriae were selected as shown in Table 1 [15,19]. The less antigenic epitopes within the dmLT were replaced with nucleotide fragments coding for these fimbriae epitopes. The epitope substitutions and the overall structure of the dmLT-MEFA protein were optimized using three-dimensional protein modeling software and visualized with the PyMOL molecular graphics system. Then, the optimized *dmlt-faeg–fanc–fasa-fedf-fim41a* chimeric gene was synthesized by Tsingke Biotechnology Co., Ltd. (Xi’an, China). Subsequently, the chimeric gene product was cloned into a pET22b(+) vector, which had been digested with specific restriction enzymes. After sequencing, the resulting plasmid was transformed into *E. coli* BL21(DE3) by chemical transformation.

### 2.3. Expression and Purification of dmLT MEFA Protein

The recombinant dmLT MEFA protein was expressed and purified. Initially, a single colony of recombinant *E. coli* BL21 (DE3) was cultured overnight in 2 × YT medium supplemented with 100 μg/mL ampicillin. The bacteria were cultured at 37 °C in 2 × YT medium supplemented with 100 μg/mL ampicillin, with vigorous shaking at 220 rpm. A 2 mL aliquot of the overnight culture was inoculated into 200 mL of fresh medium and grown until the OD600 reached 0.6. Protein expression was then induced by adding 1 mM isopropyl-β-D-1-thiogalactopyranoside (IPTG), and the culture was incubated for an additional 4 h under the same conditions. Bacterial cells were harvested by centrifugation at 12,000 rpm for 15 min. The resulting pellets were resuspended in lysis buffer and subjected to sonication for cell disruption. Inclusion bodies were collected, washed repeatedly with PBS (0.1 M, pH 7.4) containing 0.5% Tween-20, and then solubilized in 8 M urea. The solubilized protein was dialyzed against PBS and refolded using a Novagen Protein Refolding Kit according to the manufacturer’s instructions. The refolded dmLT-MEFA protein was then concentrated and stored for further purification and characterization. The recombinant protein was examined using 12% SDS-PAGE gels with chicken IgY anti-FaeG (1:8000), anti-FanC (1:2000), anti-FasA (1:1000), anti-FedF (1:5000), and anti-Fim41a (1:3000). HRP-labeled goat anti-chicken IgG (1:10,000) was used for the detection of the toxoid MEFA proteins.

### 2.4. Subcutaneous Immunization of Rabbit with dmLT MEFA

Six New Zealand white rabbits, purchased from Tongyu Farm in Taigu district, were subcutaneously immunized with 50 µg of the MEFA protein without any additional adjuvants. Following the initial immunization, four booster immunizations were administered biweekly. Seven days after the final immunization, serum samples were collected. IgG antibodies were purified using a protein G column. The animal experimental protocols involving the use of laboratory animals were reviewed and approved by the Ethics Committee of Shanxi Agricultural University (Approval No. SXAU-EAW-2022NR0292) in accordance with national and institutional guidelines for the care and use of laboratory animals.

### 2.5. Anti-Fimbriae Specific IgG Antibody Titration

To analyze the presence of antibodies specific to FaeG, FedF, FanC, FasA, and Fim41a fimbriae subunits in the serum samples obtained from immunized rabbit, an enzyme-linked immunosorbent assay (ELISA) was performed. Briefly, purified recombinant FaeG, FedF, FanC, FasA, or Fim41a protein (500 ng/mL) was immobilized to 96-well plates (Corning, Corning, NY, USA) 100 μL per well as the coating antigen overnight at 4 °C. Next day, the noncoated areas were blocked by incubation with 2% nonfat milk in PBS for 1 h at 37 °C, and the plates were washed with PBST (PBS containing 0.05% Tween 20). Next, the purified rabbit IgG (starting at 1:400 and extended to 1:25,600) was added to the respective wells. After washing, horseradish peroxidase (HRP)-conjugated goat anti-rabbit IgG (1:5000, Sigma, St. Louis, MI, USA) was used as the secondary antibody. After exposure to the 3,3′,5,5′-tetramethylbenzidine (TMB) HRP color development solution (Beyotime, Beijing, China), the optical density at 450 nm (OD450) was measured for each well, and the results were converted into antibody titers in logarithmic form (log10). The experiment was performed in triplicate.

### 2.6. Rabbit IgG Antibody Neutralization Against Cholera Toxin Enterotoxicity

LT is a homolog of commercially available cholera toxin (CT) that has been found to consistently and more effectively stimulate cyclic AMP (cAMP) in T-84 cells. Therefore, CT was used a model to evaluate purified IgG antibody for their neutralization activities against LT enterotoxicity. Briefly, 2 × 10^5^ T-84 cells were incubated with 10 ng CT that had been premixed with 30 μL of heat-inactivated (56 °C for 30 min) IgG (1 mg/mL) obtained from the immunized or control group for 3 h [16]. Following incubation, cells were washed thoroughly with PBS to remove extracellular cAMP and then lysed with 0.1 M HCl containing 0.5% Triton X-100 to release intracellular cAMP for subsequent quantification. The intracellular cAMP levels in T-84 cells were quantified using a commercial ELISA kit (Enzo Life Sciences, Farmingdale, NY, USA) according to the manufacturer’s instructions. Cells incubated with CT alone (without serum) served as the positive control, while cells treated with cell culture medium alone (lacking both toxin and serum) were used as the baseline cAMP control.

### 2.7. Rabbit IgG-Mediated Inhibition of K88+, F18+, K99+, 987P+, and F41+ ETEC Strain Adhesion to IPEC-J2

The in vitro antibody-mediated adhesion inhibition assay was conducted using porcine small intestinal epithelial cell line IPEC-J2 and wild-type ETEC strains expressing K88, F18, K99, 987P, or F41 fimbriae, following established protocols [15]. For the assay, ETEC bacteria were cultured to the logarithmic growth phase, harvested, and resuspended in sterile phosphate-buffered saline (PBS) to achieve the desired inoculum concentration. The bacterial suspensions, with a multiplicity of infection (MOI) of 5 bacteria per cell, were co-incubated with 30 µL of purified IgG (1 mg/mL) from each experimental group on a shaker with gentle agitation for 30 min at room temperature. Subsequently, the IgG/bacteria mixture was added to individual wells of a 12-well tissue culture plate containing a confluent monolayer of IPEC-J2 cells. The IPEC-J2 cells, along with the IgG-bacteria mixture, were incubated at 37 °C in a 5% CO_2_ atmosphere for 1 h to allow bacterial adhesion. After incubation, the cells were gently washed with sterile PBS to remove non-adherent bacteria. Adherent ETEC bacteria were then released by lysing the infected cells with 0.5% Triton X-100. The resulting lysate, containing the intracellular and surface-adherent bacteria, was serially diluted in PBS and plated onto LB agar plates for colony counting. The LB agar plates were incubated overnight at 37 °C and the number of bacterial colonies (colony-forming units, CFU) was counted.

### 2.8. Statistical Analysis

Rabbit IgG titers were expressed as logarithmic values (log10). The in vitro rabbit IgG-mediated adhesion inhibition assay was performed in triplicate, and data were analyzed using GraphPad Prism version 6.0 (GraphPad Software, La Jolla, CA, USA). Differences between treatment groups were assessed by Student’s *t*-test, and results are presented as mean values ± standard deviations. Statistical significance was set at *p* < 0.05. Statistical significance was determined by a *p* value of less than 0.05.

## 3. Results

### 3.1. dmLT MEFA Carried the Epitopes of the Five Fimbriae Subunits of ETEC

**Figure 1:**
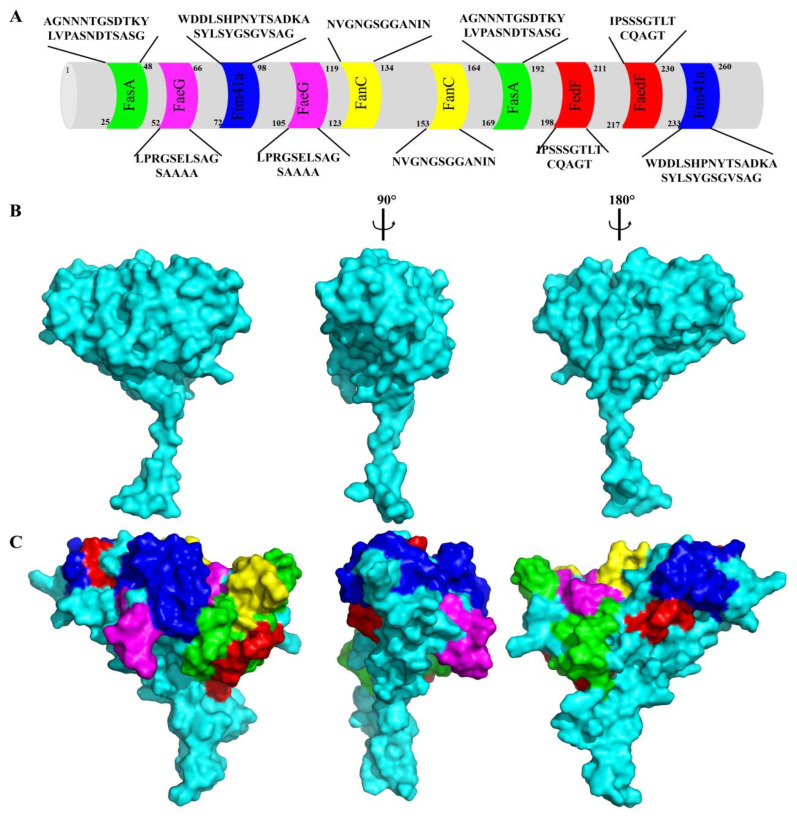
The structure of LTa1 and MEFA. (**A**) The sequence of dmLT MEFA; (**B**) the structure of LTa (PDB ID: 1LTA); (**C**) the structure of the predicted MEFA. Epitopes of the adhesive subunits of the five fimbriae are highlighted in different colors: FasA (green), FaeG (pink), Fim41a (blue), FanC (yellow), and FedF (red).

The neutralizing epitopes of K88+ (LPRGSELSAGSAAAA), K99+ (NVGNGSGGANIN), 987P+ (AGNNNTGSDTKYLVPASNDTSASG), F41+ (WDDLSHPNYTSADKASYLSYGSGVSAG), and F18+ (IPSSSGTLTCQAGT) ETEC fimbriae were selected based on their values of protective antigen prediction, which were greater than 0.4 (refer to Table 1). Ten surface-exposed loop regions of the dmLT B-subunit were selected as insertion sites for ETEC epitopes, based on structural accessibility and sequence variability analysis (Figure 1A), aiming to minimize disruption of dmLT’s structural integrity and adjuvant function. This substitution resulted in the construction of chimeric genes, specifically *dmlt-fase-faeg-fim41a-fanc-fedf* (as shown in Figure 1A). Subsequently, a total of 30 structural models were generated for the dmLT-MEFA protein. The model with the highest conformer score displayed a three-dimensional structure closely resembling the native dmLT backbone (PDB ID: 1LTA), as shown in Figure 1B. The models’ results indicated that all the selected epitopes from the FaeG, FanC, FasA, Fim41a, and FedF fimbriae were exposed on the surface of the MEFA protein (as depicted in Figure 1C).

### 3.2. The Fimbriae Epitopes Displayed on dmLT MEFA

**Figure 2:**
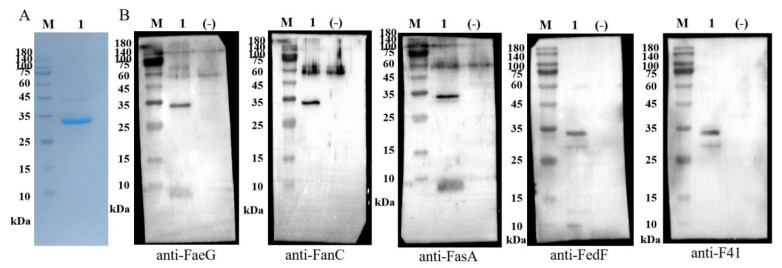
Expression and identification of MEFA. (**A**) Expression of MEFA recombinant protein; Line M, protein Marker; Line 1, MEFA. (**B**) Identification of fimbriae exposed on dmLT MEFA; Line M, protein marker; Line 1, dmLT MEFA; Line (-), dmLT without fimbria epitopes.

Tag is considered less desirable for vaccine development due to the potential induction of anti-tag antibodies [15]. For instance, anti-6His tag antibodies could be induced by antigen with His-tag. Anti-histidine immunity may pose health risk, as histidine is an essential amino acid for maintaining good health. Therefore, in this study, the toxoid dmLT MEFA was not tagged with any additional sequences. The dmLT MEFA protein was expressed as inclusion bodies in E. coli, which were then washed and dissolved in 8 M urea. The inclusion bodies were purified by sequential centrifugation steps, and the resulting pellet was solubilized in 8 M urea, followed by affinity chromatography under denaturing conditions. Subsequent dialysis was carried out in PBS to remove residual urea, and the refolded protein was concentrated and purified. Purity was assessed by the densitometry of Coomassie-stained gels, showing >90% purity, and protein concentration was determined using the BCA assay with a denaturant-compatible standard curve. The final yield was approximately 2.5 mg per liter of culture. The expected protein band at 33 kDa was observed (Figure 2A). Furthermore, the dmLT MEFA proteins were recognized by specific anti-FaeG, anti-FanC, anti-FasA, anti-Fim41a, and anti-FedF IgY antibodies (Figure 2B).

### 3.3. Rabbit IgG Response to All Five Fimbriae Adhesive Subunits

**Figure 3:**
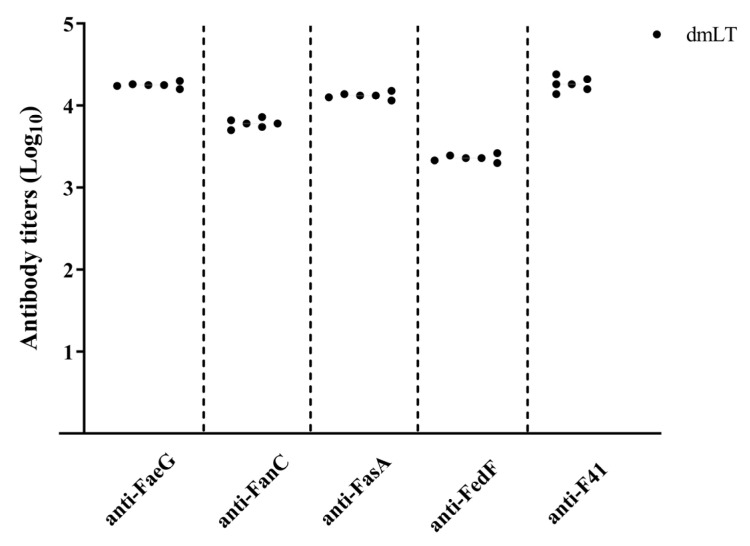
Rabbit IgG titers for ETEC FaeG, FanC, FasA, Fim41a, and FedF fimbria recombinant protein.

DmLT is known as a potent adjuvant for developing specific antibody responses comparable to other adjuvants [21,26]. Serum IgG from mice immunized with the dmLT adjuvant demonstrated significantly enhanced neutralizing activity against CT compared to controls, indicating the adjuvant’s ability to elicit functional, toxin-neutralizing antibodies [21]. Therefore, in this study, New Zealand rabbits were subcutaneously immunized with toxoid dmLT MEFA recombinant proteins without other adjuvants. After the final immunization, serum was collected to isolate IgG using a protein G column. ELISAs were performed using recombinant FaeG, FanC, FasA, FedF, and Fim41a as coating antigens. The results indicated that the IgG titers in immunized rabbits were 4.25 ± 0.03, 3.78 ± 0.06, 4.12 ± 0.04, 3.36 ± 0.05, and 4.26 ± 0.08 (reported as log10 transformed results) (Figure 3). Adjuvants can significantly modulate various aspects of the immune response, including the magnitude and avidity of antibody responses to vaccine antigens. Notably, no antibodies against the fimbrial subunit antigens were detected in the serum samples from control rabbits, confirming the specificity of the observed immune response to vaccination. The results suggested that dmLT MEFA recombinant protein could elicit antibodies specific to the five fimbrial adhesive subunit proteins.

### 3.4. Rabbit IgG Antibody Neutralized CT In Vitro

**Figure 4:**
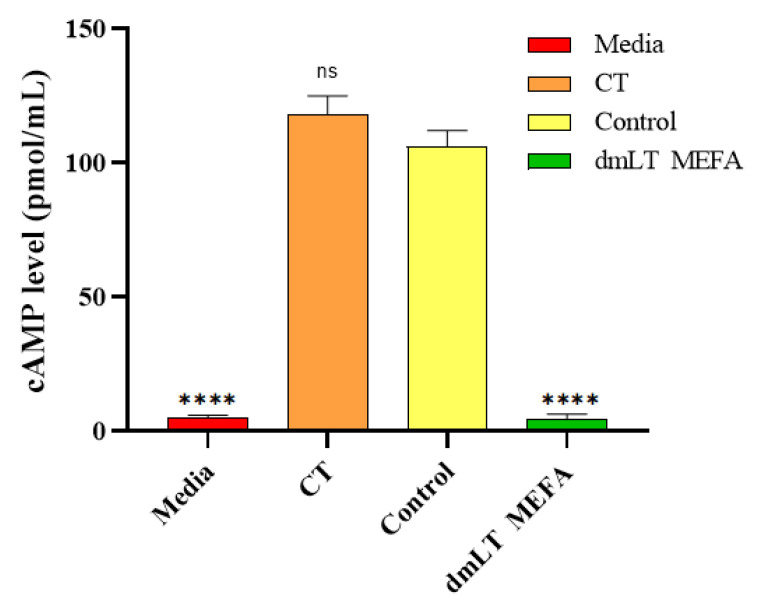
Rabbit IgG in vitro neutralization activity against CT. T-84 cells and cAMP ELISA kit were used to measure IgG neutralizing activities against CT. Columns and bars represent the means and standard deviations of cAMP levels. ****, *p* < 0.0001.

The IgG from rabbits displayed neutralization activities against the enterotoxicity of CT, as shown in Figure 4. When T-84 cells were incubated with 10 ng CT, the cAMP levels were 118 ± 3.16 pmol/mL. These levels were significantly different from the cAMP levels in T-84 cells incubated with 10 ng CT and the IgG from the immunization group (4.33 ± 0.23 pmol/mL; *p* < 0.001). The cAMP level in cells incubated with 10 ng CT and the serum samples of the control rabbit was 106 ± 2.1 pmol/mL. The baseline cAMP level in T-84 cells was 5.0 ± 0.14 pmol/mL (Figure 4). This suggested that the dmLT MEFA protein could elicited neutralizing antibodies for CT.

### 3.5. Serum Samples from Immunized Rabbits Inhibited Adherence to IPEC-J2 of ETEC Bacteria Expressing the Corresponding Fimbriae

**Figure 5:**
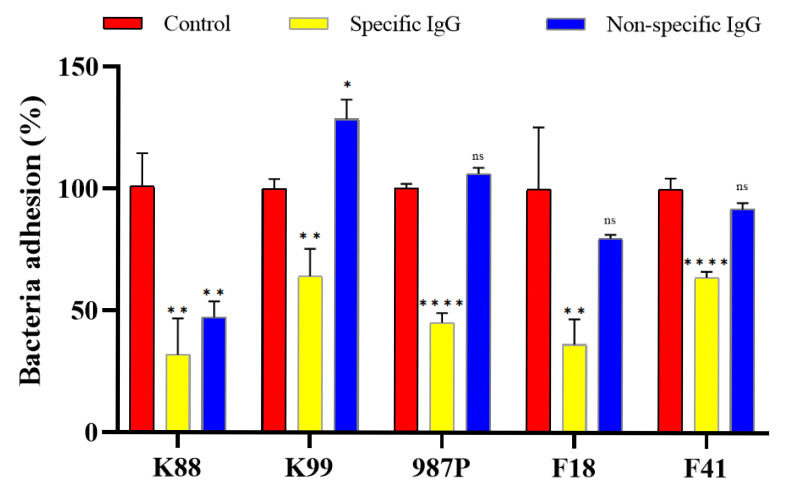
IgG-inhibited adherence of K88, K99, 987P, F41, and F18 strains to IPEC-J2 cell lines. ETEC after incubated with rabbit serum samples pooled from each immunization group (n = 6) or the control and non-specific group, were transferred to IPEC-J2 cells. Incubated for 1 h, cells were washed to remove non-adherent bacteria and lysed. Adherent bacteria were collected, diluted, and plated on LB agar plates. Bacteria were counted for CFUs after overnight growth at 37 °C. The number of adherent bacteria in the control group was referred as 100%. * indicate p value of <0.01. ns means no significant. ** and **** indicate *p* value of <0.01 and <0.0001, respectively.

Previous studies have shown that the porcine intestinal epithelial cell lines IPEC-J2 serves as suitable in vitro models for studying the adherence of porcine ETEC [23,24]. The results of our study demonstrated that ETEC bacteria expressing FaeG, FanC, FasA, FedF, or Fim41a recombinant proteins, when incubated with IgG from immunized rabbit, exhibited significantly reduced adherence to IPEC-J2 cell lines compared to ETEC strains incubated with serum from control (Figure 5) (*p* < 0.01). When ETEC strains expressing FaeG, FanC, FasA, FedF, or Fim41a fimbriae were pre-incubated with rabbit IgG specific to the dmLT toxoid-based MEFA vaccine (Figures S1–S5), they exhibited approximately 63%, 74%, 71%, 70%, and 63% reduction in adherence to IPEC-J2 cells, respectively, compared to strains treated with control IgG (Figure 5). These results indicate that the MEFA-induced antibodies effectively block the attachment of diverse ETEC fimbrial types, demonstrating the vaccine’s broad-spectrum anti-adhesive activity. In addition, non-specific IgG showed no inhibition activity against all tested fimbriae except for K88 fimbriae (Figure 5). One possible explanation for this observation is that the non-specific IgG preparation used in the study might contain trace amounts of antibodies that have some affinity for K88 fimbriae. Although these antibodies are not specifically designed to target K88, they might have arisen during the production process due to exposure to environmental antigens that share structural similarities with K88 fimbriae. This cross-reactivity could account for the observed inhibition activity. Another hypothesis is that the binding sites on the K88 fimbriae might have a more generalized structure compared to those of the other fimbriae tested. Such a structure could potentially interact with the constant region of IgG molecules, leading to nonspecific binding and inhibition of adhesion. The results suggested that the antibodies elicited by toxoid dmLT MEFA chimeric multiepitope could block the ETEC expressing FaeG, FanC, FasA, FedF, or Fim41a attachment to porcine intestine.

## 4. Discussion

ETEC-associated diarrhea is a major problem in piglets, causing high mortality and morbidity in the swine industry worldwide. One of the main factors contributing to this disease is the attachment of ETEC to specific receptors in the pig intestinal epithelial cells, facilitated by fimbrial adhesins. This allows the bacteria to colonize in the small intestine, leading to the initiation of the disease [2]. Following colonization, ETEC strains secrete enterotoxins including LT and ST to disrupt fluid homeostasis, resulting in diarrhea. Therefore, fimbriae are the main virulence factors associated with ETEC and have been targeted for the development of prevention strategies.

ETEC strains have been isolated from cases of neonatal and post-waning piglet’s diarrhea expresses at least five fimbrial adhesins and toxins [27]. Although the virulence determinants and pathogenic mechanisms underlying ETEC-mediated diarrhea in neonatal and post-weaning piglets are well understood, few effective vaccines are currently available to prevent this disease, highlighting a critical need for improved immunoprophylactic strategies [28]. MEFA technology is a structure-based vaccine development technique that combines computational biology and structural biology. This approach has been successful in developing multivalent vaccines against heterogeneous virulence factors [18,19,20]. In this study, dmLT was utilized as a molecular scaffold to design a fimbria-targeted MEFA vaccine, incorporating duplicated neutralizing epitopes from five fimbrial subunits—FaeG (K88), FedF (F18), FanC (K99), FasA (987P), and Fim41a (F41)—expressed by ETEC strains associated with diarrhea in piglets. This multivalent design aims to elicit broad protective immunity against the major adhesins involved in intestinal colonization. The inclusion of duplicate epitopes aims to increase the valency of the antigen-presenting units. By presenting multiple copies of the same epitope, the likelihood of T-cell receptor engagement will be enhanced, potentially leading to a stronger and more robust immune response. Multiple copies of an epitope can increase the probability that T-cells will encounter the antigen, thus improving the chances of activation and proliferation of antigen-specific T-cells. In addition, redundancy in epitope presentation can help overcome potential issues related to antigen processing and presentation variability. Different cells in the body may process and present antigens differently; having multiple copies of the same epitope ensures that even if some copies are lost during processing, others remain available for presentation via MHC molecules. Computational modeling revealed that all selected epitopes were successfully displayed on the surface of the MEFA protein, ensuring their accessibility to the immune system and supporting the rational design of a highly immunogenic, multivalent vaccine candidate. After expression of the dmLT MEFA, each epitope was recognized by antibodies specific to its corresponding fimbria. Immunization of rabbits with the dmLT MEFA proteins resulted in the production of high levels of antibodies specific to each fimbria. Epitopes from FaeG (K88) and FedF (F18)—chosen for their high antigenicity and surface exposure—elicited strong IgG titers and effective adhesion inhibition, those from FanC (987P) and Fim41a (F41) induced lower-than-expected antibody levels and neutralizing capacity despite similar antigenicity predictions. This discrepancy may arise from structural constraints within the dmLT-MEFA fusion that affect epitope accessibility, competition among multiple epitopes for B-cell recognition, variations in T-cell help, or differences in assay sensitivity due to receptor expression levels on target cells. This reflection enhances our understanding of the vaccine’s immunological profile and guides further refinement of the MEFA platform.

Studies have demonstrated that both a CFA/I/II/IV MEFA, carrying epitopes of the primary subunits of the seven most significant human ETEC adhesins [29], and another form of CFA adhesin tip, MEFA [30], which carried the epitopes of the tip subunit of nine crucial human ETEC adhesins, were capable of inducing protective antibodies that prevented the adherence of *E. coli* strains to Caco-2 cells expressing these adhesins. Neutralizing epitopes from FaeG, FedF, FasA, Fim41a, and FanC were, respectively, inserted into the backbones of FedF or FaeG, generating MEFA recombinant proteins. These proteins can elicit specific antibodies that effectively block the adhesion of ETEC strains expressing K88, F18, K99, 987P, and F41 fimbriae to porcine intestinal epithelial cell lines IPEC-J1 and IPEC-J2 [18,19]. Likewise, in this study, our findings demonstrate that antibodies from immunized groups effectively inhibited the adhesion of porcine ETEC strains expressing K88, F18, K99, 987P, and F41 fimbriae to IPEC-J2 cells, confirming the functional activity and broad neutralizing capacity of the induced immune response. Additionally, the antibodies were able to neutralize the enterotoxin CT in vitro. Therefore, the designed dmLT MEFAs showed promise in developing broadly protective vaccines against ETEC strains expressing K88+, K99+, 987P+, F41+, and F18+ fimbriae. Overall, the study reveals the potential of using MEFA technology to develop effective vaccines against ETEC-associated diarrhea in piglets. While our dmLT-MEFA construct addresses antigenic diversity, future work must confront additional barriers to effective neonatal vaccination, including maternal antibody interference and the need for robust mucosal immunity. Alternative delivery routes or prime-boost strategies may be required to overcome these challenges and elicit protective immunity in early life.

## 5. Conclusions

In this study, we developed a fimbriae-targeted MEFA protein based on dmLT backbone to incorporate neutralizing epitopes of ETEC (FaeG, FedF, FanC, FasA, and Fim41a). Both the computational modeling and experimental findings confirmed that all relevant epitopes were prominently displayed on the surface of the MEFA. Subsequent administration of MEFA proteins via the subcutaneous route in rabbits resulted in the generation of antibodies capable of cross-reacting with K88, K99, 987P, F18, and F41 fimbriae, with IgG titers of 4.25 ± 0.03, 3.78 ± 0.06, 4.12 ± 0.04, 3.36 ± 0.05, and 4.26 ± 0.08. In addition, the dmLT MEFA protein could elicited neutralizing antibodies for CT. These antibodies also demonstrated significant inhibition of ETEC strains adherence to porcine small intestinal epithelial cells. These exciting results suggested that the fimbriae-targeted MEFA could be a promising vaccine candidate for forming a multivalent antigen to induce broadly protective immunity.

## Figures and Tables

**Table 1:** The antigenic value of selected ETEC fimbrial peptides.

ETEC Strain	Fimbrial Protein	Selective Neutralizing Peptides	Predictive Value ^a^
K88	FaeG	LPRGSELSAGSAAAA	0.6044
K99	FanC	NVGNGSGGANIN	2.3671
987P	FasA	AGNNNTGSDTKYLVPASNDTSASG	1.1994
F41	Fim41a	WDDLSHPNYTSADKASYLSYGSGVSAG	0.5260
F18	FedF	IPSSSGTLTCQAGT	0.9849

^a^ The peptides was selected as neutralizing peptides, and the default threshold of protective antigen prediction was 0.4, which was predicted by Vaxijen 2.0.

## Data Availability

The original contributions presented in this study are included in this article, further inquiries can be directed to the corresponding author.

## Supplementary Materials

The following supporting information can be downloaded at: https://www.mdpi.com/article/10.3390/ani15192858/s1, Figure S1: IgG inhibits K88 strain adhension IPEC-J2 at 100-fold, Figure S2: IgG inhibits K99 strain adhension to IPEC-J2 cells at 100-fold, Figure S3: IgG inhibits 987P strain adhension to IPEC-J2 cells at 100-fold, Figure S4: IgG inhibits F18 strain adhension to IPEC-J2 cells at 100-fold, Figure S5: IgG inhibits F41 strain adhension to IPEC-J2 cells at 100-fold.

## Abbreviations

The following abbreviations are used in this manuscript:

ETEC	Enterotoxigenic Escherichia coli
dmLT	Double-mutant heat-labile toxin
MEFA	Multivalent epitope fusion antigens
LT	Heat-labile toxin
ST	Heat-stable toxins
CFA	Colonization factor antigen
IPTG	Isopropyl-β-D-1-thiogalactoside
ELISA	Enzyme-linked immunosorbent assay
CFU	Colony-forming units

## References

[1] Seo H., Zhang W. (2020). Development of effective vaccines for enterotoxigenic *Escherichia coli*. Lancet Infect. Dis..

[2] Gaastra W., de Graaf F.K. (1982). Host-specific fimbrial adhesins of noninvasive enterotoxigenic *Escherichia coli* strains. Microbiol Rev..

[3] Guiral E., Gonçalves Quiles M., Muñoz L., Moreno-Morales J., Alejo-Cancho I., Salvador P., Alvarez-Martinez M.J., Marco F., Vila J. (2019). Emergence of resistance to quinolones and β-lactam antibiotics in enteroaggregative and enterotoxigenic *Escherichia coli* causing traveler’s diarrhea. Antimicrob. Agents Chemother..

[4] Frost I., Van Boeckel T.P., Pires J., Craig J., Laxminarayan R. (2019). Global geographic trends in antimicrobial resistance: The role of international travel. J. Travel Med..

[5] von Mentzer A., Svennerholm A.M. (2024). Colonization factors of human and animal-specific enterotoxigenic *Escherichia coli* (ETEC). Trends Microbiol..

[6] Zhang W., Sack D.A. (2012). Progress and hurdles in the development of vaccines against enterotoxigenic *Escherichia coli* in humans. Expert Rev. Vaccines.

[7] Seo H., Garcia C., Ruan X., Duan Q., Sack D.A., Zhang W. (2021). Preclinical characterization of immunogenicity and efficacy against diarrhea from MecVax, a multivalent enterotoxigenic *E. coli* vaccine candidate. Infect. Immun..

[8] Zhang W., Sack D.A. (2015). Current progress in developing subunit vaccines against enterotoxigenic *Escherichia coli* (ETEC) associated diarrhea. Clin. Vaccine Immunol..

[9] Walker R., Dull P. (2017). Combination vaccine strategies to prevent enteric infections. Vaccine.

[10] Fleckenstein J.M. (2021). Confronting challenges to enterotoxigenic *Escherichia coli* vaccine development. Front. Trop. Dis..

[11] Upadhyay I., Lauder K.L., Li S., Ptacek G., Zhang W. (2022). Intramuscularly administered enterotoxigenic *Escherichia coli* (ETEC) vaccine candidate MecVax prevented H10407 intestinal colonization in an adult rabbit colonization model. Microbiol. Spectr..

[12] Walker R.I., Bourgeois A.L. (2023). Oral inactivated whole cell vaccine for mucosal immunization: ETVAX case study. Front. Immunol..

[13] Tobias J., Svennerholm A.M. (2012). Strategies to overexpress enterotoxigenic *Escherichia coli* (ETEC) colonization factors for the construction of oral whole-cell inactivated ETEC vaccine candidates. Appl. Microbiol. Biotechnol..

[14] Borde A., Ekman A., Larsson A., Carlin N., Holmgren J., Tobias J. (2016). Preparation and preclinical evaluation of a freeze-dried formulation of a novel combined multivalent whole-cell/B-subunit oral vaccine against enterotoxigenic *Escherichia coli* diarrhea. Eur. J. Pharm. Biopharm..

[15] Lu T., Moxley R.A., Zhang W. (2020). Application of a novel epitope- and structure-based vaccinology-assisted fimbria-toxin multiepitope fusion antigen of enterotoxigenic *Escherichia coli* for development of multivalent vaccines against porcine postweaning diarrhea. Appl. Environ. Microbiol..

[16] Duan Q., Lu T., Garcia C., Yañez C., Nandre R.M., Sack D.A., Zhang W. (2018). Co-administered tag-less toxoid fusion 3×STaN12S-mnLTR192G/L211A and CFA/I/II/IV MEFA (multiepitope fusion antigen) induce neutralizing antibodies to 7 adhesins (CFA/I, CS1-CS6) and both enterotoxins (LT, STa) of enterotoxigenic *Escherichia coli* (ETEC). Front. Microbiol..

[17] Leach S., Lundgren A., Carlin N., Löfstrand M., Svennerholm A.M. (2017). Cross-reactivity and avidity of antibody responses induced in humans by the oral inactivated multivalent enterotoxigenic *Escherichia coli* (ETEC) vaccine ETVAX. Vaccine.

[18] Duan Q., Pang S., Wu W., Jiang B., Zhang W., Liu S., Wang X., Pan Z., Zhu G. (2020). A multivalent vaccine candidate targeting enterotoxigenic *Escherichia coli* fimbriae for broadly protecting against porcine post-weaning diarrhea. Vet. Res..

[19] Duan Q., Wu W., Pang S., Pan Z., Zhang W., Zhu G. (2020). Coimmunization with two enterotoxigenic *Escherichia coli* (ETEC) fimbrial multiepitope fusion antigens induces the production of neutralizing antibodies against five ETEC fimbriae (F4, F5, F6, F18, and F41). Appl. Environ. Microbiol..

[20] Li S., Anvari S., Ptacek G., Upadhyay I., Kaminski R.W., Sack D.A., Zhang W. (2023). A broadly immunogenic polyvalent *Shigella* multiepitope fusion antigen protein protects against *Shigella sonnei* and *Shigella flexneri* lethal pulmonary challenges in mice. Infect. Immun..

[21] Molina Estupiñan J.L., Aradottir Pind A.A., Foroutan Pajoohian P., Jonsdottir I., Bjarnarson S.P. (2023). The adjuvants dmLT and mmCT enhance humoral immune responses to a pneumococcal conjugate vaccine after both parenteral or mucosal immunization of neonatal mice. Front. Immunol..

[22] Stone A.E., Rambaran S., Trinh I.V., Estrada M., Jarand C.W., Williams B.S., Murrell A.E., Huerter C.M., Bai W., Palani S. (2023). Route and antigen shape immunity to dmLT-adjuvanted vaccines to a greater extent than biochemical stress or formulation excipients. Vaccine.

[23] Nandre R.M., Duan Q., Wang Y., Zhang W. (2017). Passive antibodies derived from intramuscularly immunized toxoid fusion 3xSTaN12S-dmLT protect against STa+ enterotoxigenic *Escherichia coli* (ETEC) diarrhea in a pig model. Vaccine.

[24] Nandre R., Ruan X., Lu T., Duan Q., Sack D., Zhang W. (2018). Enterotoxigenic *Escherichia coli* adhesin-toxoid multiepitope fusion antigen CFA/I/II/IV-3×STaN12S-dmLTR192G/L211A-derived antibodies inhibit adherence of seven adhesins, neutralize enterotoxicity of LT and STa toxins, and protect piglets against diarrhea. Infect. Immun..

[25] Das S., Howlader D.R., Zheng Q., Ratnakaram S.S.K., Whittier S.K., Lu T., Keith J.D., Picking W.D., Birket S.E., Picking W.L. (2020). Development of a broadly protective, self-adjuvanting subunit vaccine to prevent infections by *Pseudomonas aeruginosa*. Front. Immunol..

[26] Liang H., Poncet D., Seydoux E., Rintala N.D., Macie M., Ruiz S., Orr M.T. (2019). The TLR4 agonist adjuvant SLA-SE promotes functional mucosal antibodies against a parenterally delivered ETEC vaccine. NPJ Vaccines.

[27] Duan Q.D., Yao F.H., Zhu G.Q. (2012). Major virulence factors of enterotoxigenic *Escherichia coli* in pigs. Ann. Microbiol..

[28] Fairbrother J.M., Nadeau E., Gyles C.L. (2005). *Escherichia coli* in postweaning diarrhea in pigs: An update on bacterial types, pathogenesis, and prevention strategies. Anim. Health Res. Rev..

[29] Ruan X., Knudsen D.E., Wollenberg K.M., Sack D.A., Zhang W. (2014). Multi-epitope fusion antigen induces broadly protective antibodies that prevent adherence of *Escherichia coli* strains expressing colonization factor antigen I (CFA/I), CFA/II, and CFA/IV. Clin. Vaccine Immunol..

[30] Nandre R.M., Ruan X., Duan Q., Sack D.A., Zhang W. (2016). Antibodies derived from an enterotoxigenic *Escherichia coli* (ETEC) adhesin tip MEFA (multi-epitope fusion antigen) against adherence of nine ETEC adhesins: CFA/I, CS1, CS2, CS3, CS4, CS5, CS6, CS21 and EtpA. Vaccine.

