# Supplementary material for: Chimeric Fimbrial Multiepitope Antigen Fused to Double-Mutant LT (dmLT) Induces Antibodies That Inhibit Enterotoxigenic E. coli Adhesion in Porcine IPEC-J2 Cells
Source: Animals (Basel). 2025 Sep 30;15(19):2858. doi: 10.3390/ani15192858 (PMC12523420; doi:10.3390/ani15192858)

Supplementary materials

Chimeric Fimbrial Multiepitope Antigen Fused to Double-Mutant LT (dmLT) Induces Antibodies That Inhibit Enterotoxigenic *E. coli* Adhesion in Porcine IPEC-J2 Cells

Jinxin He, Hongrui Liu, Yuexin Li, Jiashu Chang, Yayun Yang and Shaopeng Gu *

College of Veterinary Medicine, Shanxi Agricultural University, Taigu, Shanxi 030801, PR China.

*Address correspondence to:

Dr. Shaopeng Gu, Tel: +86 0354-6289229; E-mail: shpgu@163.com.

Fig. S1. IgG inhibits K88 strain adhension IPEC-J2 at 100-fold. (a) Untreated IPEC-J2 cells; (b) Incubate 5×10^5 of K88 bacteria suspension with 30 μL sterile PBS for 30 min to adhesion to IPEC-J2 cells for 2 h; (c) Incubate 5×10^5 of K88 bacteria suspension with 30 μL of specific IgG for 30 min to adhesion to IPEC-J2 cells for 2 h; (d) Incubate 5×10^5 of K88 bacteria suspension with 30 μL of non-specific IgG 30 min to adhesion to IPEC-J2 cells for 2 h.

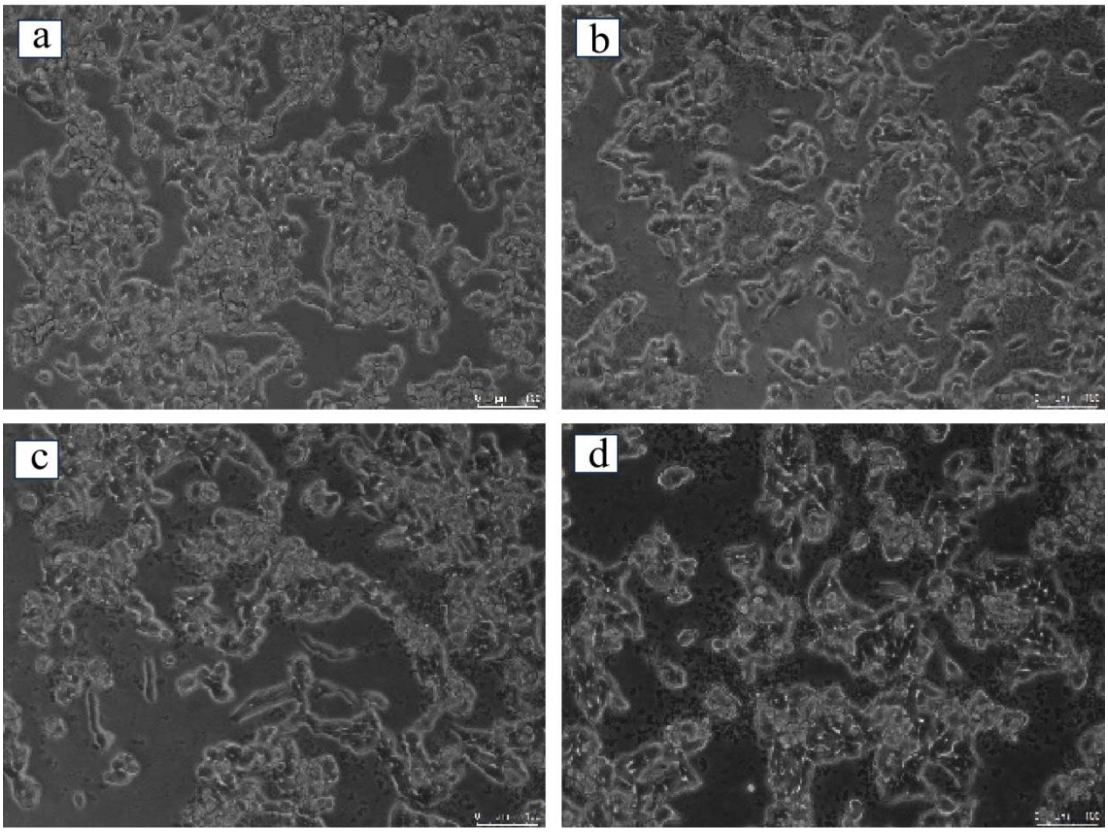

Fig. S2. IgG inhibits K99 strain adhension to IPEC-J2 cells at 100-fold. (a) Untreated IPEC-J2 cells; (b) Incubate 5×10^5 of K99 bacteria suspension with 30 μL sterile PBS for 30 min to adhesion to IPEC-J2 cells for 2 h; (c) Incubate 5×10^5 of K99 bacteria suspension with 30 μL of specific IgG for 30 min to adhesion to IPEC-J2 cells for 2 h; (d) Incubate 5×10^5 of K99 bacteria suspension with 30 μL of non-specific IgG 30 min to adhesion to IPEC-J2 cells for 2 h.

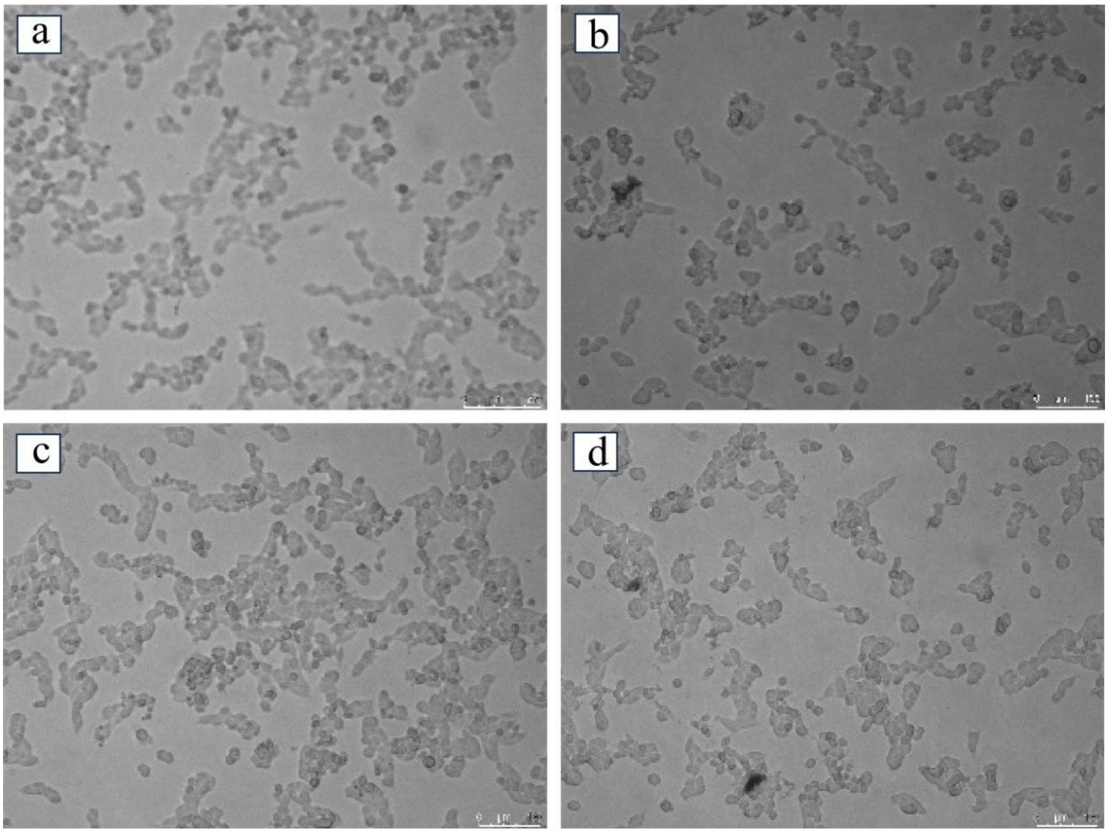

Fig. S3. IgG inhibits 987P strain adhension to IPEC-J2 cells at 100-fold. (a) Untreated IPEC-J2 cells; (b) Incubate 5×10^5 of 987P bacteria suspension with 30 μL sterile PBS for 30 min to adhesion to IPEC-J2 cells for 2 h; (c) Incubate 5×10^5 of 987P bacteria suspension with 30 μL of specific IgG for 30 min to adhesion to IPEC-J2 cells for 2 h; (d) Incubate 5×10^5 of 987P bacteria suspension with 30 μL of non-specific IgG 30 min to adhesion to IPEC-J2 cells for 2 h.

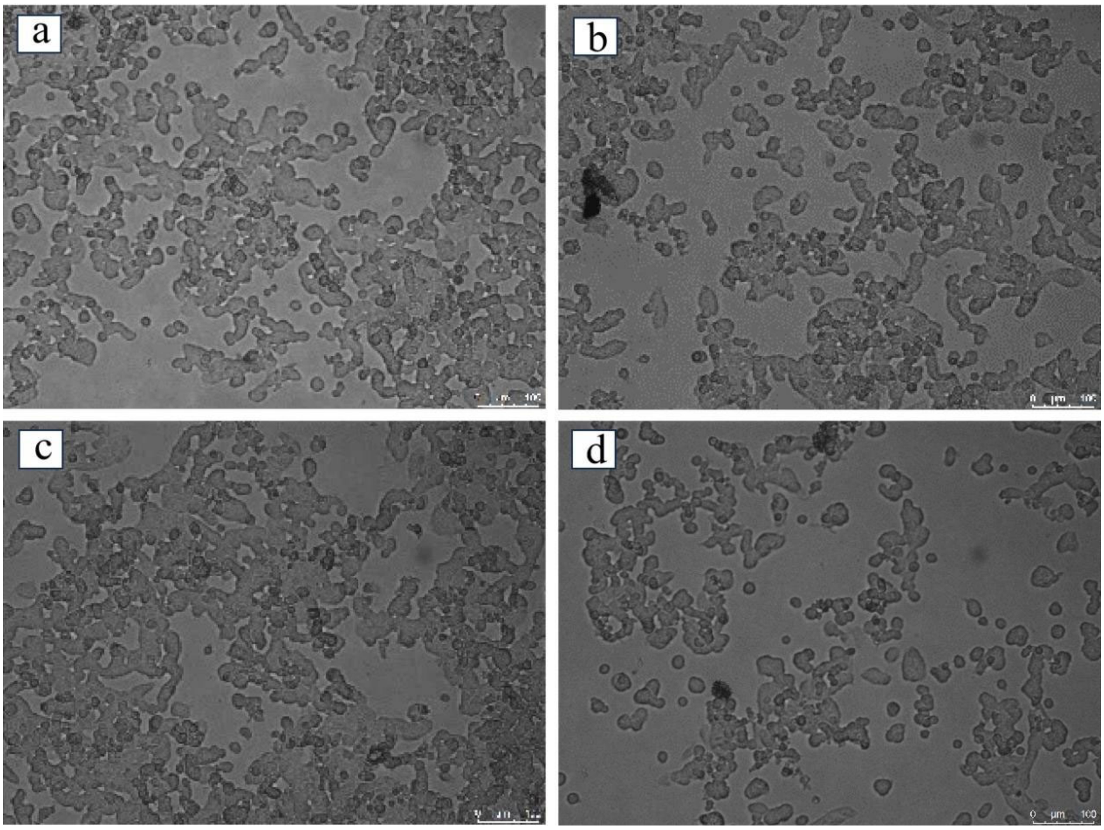

Fig. S4. IgG inhibits F18 strain adhension to IPEC-J2 cells at 100-fold. (a) Untreated IPEC-J2 cells; (b) Incubate 5×10^5 of F18 bacteria suspension with 30 μL sterile PBS for 30 min to adhesion to IPEC-J2 cells for 2 h; (c) Incubate 5×10^5 of F18 bacteria suspension with 30 μL of specific IgG for 30 min to adhesion to IPEC-J2 cells for 2 h; (d) Incubate 5×10^5 of F18 bacteria suspension with 30 μL of non-specific IgG 30 min to adhesion to IPEC-J2 cells for 2 h.

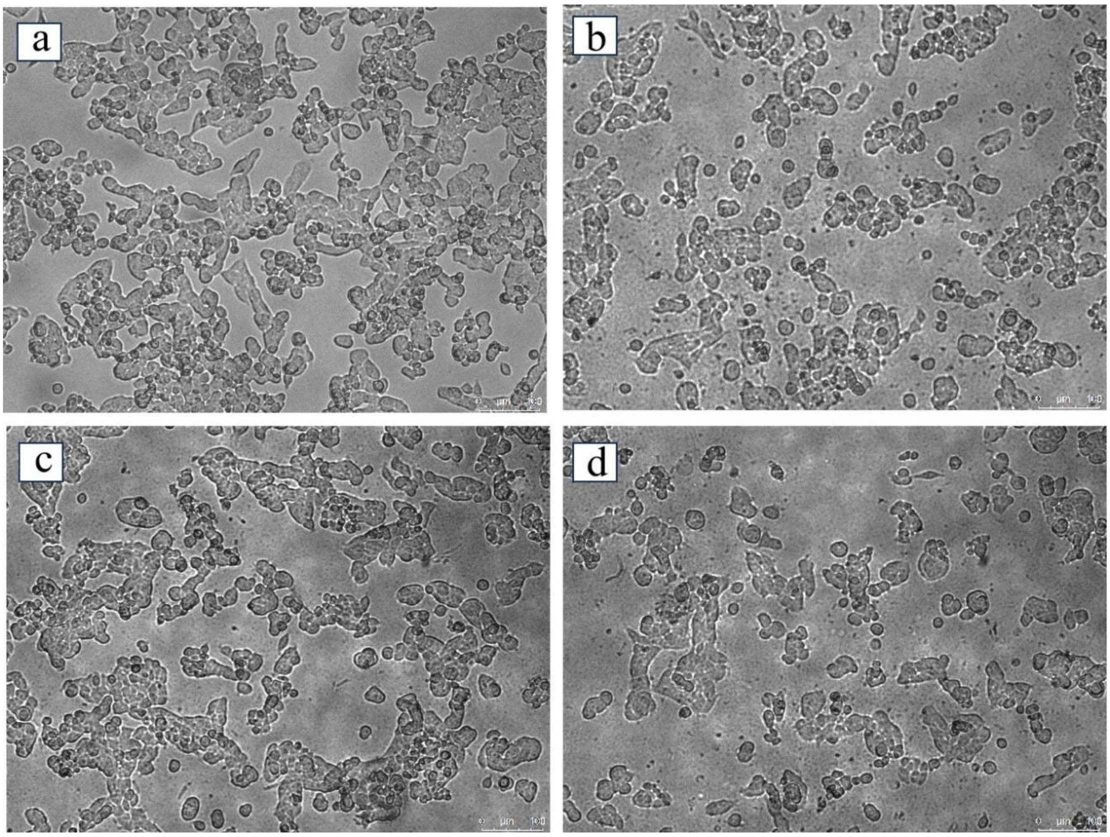

Fig. S5. IgG inhibits F41 strain adhension to IPEC-J2 cells at 100-fold. (a) Untreated IPEC-J2 cells; (b) Incubate 5×10^5 of F41 bacteria suspension with 30 μL sterile PBS for 30 min to adhesion to IPEC-J2 cells for 2 h; (c) Incubate 5×10^5 of F41 bacteria suspension with 30 μL of specific IgG for 30 min to adhesion to IPEC-J2 cells for 2 h; (d) Incubate 5×10^5 of F41 bacteria suspension with 30 μL of non-specific IgG 30 min to adhesion to IPEC-J2 cells for 2 h.

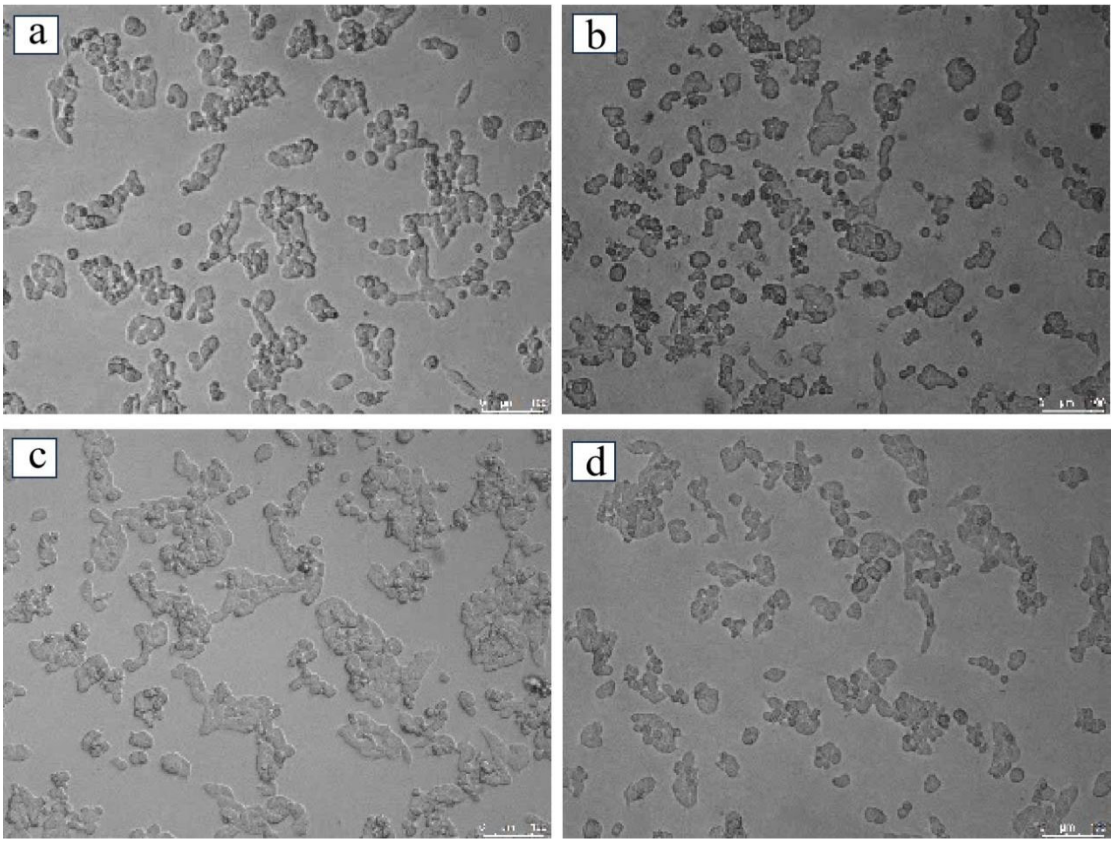

Supplement: Supplementary file 1 [file animals-15-02858-s001.zip › animals-3846457-supplementary.pdf]
